# Mendelian Randomisation Analysis of Dietary Exposures and Potential Risks of Anxiety and Depression

**DOI:** 10.62641/aep.v53i5.1969

**Published:** 2025-10-05

**Authors:** Xiaoling Yu, Runxia Hang, Qian Gan, Juanjuan Peng

**Affiliations:** ^1^Department of Outpatient, General Hospital of Eastern Theater Command, 210002 Nanjing, Jiangsu, China

**Keywords:** mendelian randomisation analysis, anxiety, depression, diet, mental health

## Abstract

**Background:**

Anxiety and depression are widespread mental health disorders with substantial global influence. dietary exposures have been proposed as modifiable risk factors of these diseases, but their causal relationships remain uncertain. This study aimed to elucidate the causal effects of specific dietary exposures on the risks of anxiety and depression using Mendelian randomisation (MR).

**Methods:**

Two-sample MR analysis was performed using summary-level data from large-scale genome-wide association studies of European populations. Nineteen dietary exposures, including beef, cereals, tea, non-oily fish and unsalted peanuts, were analysed. Causal estimates were obtained using the inverse variance weighted (IVW) method, MR–Egger regression and weighted median approach. Sensitivity analyses were conducted to assess heterogeneity and horizontal pleiotropy.

**Results:**

High consumption of beef (odds ratio [OR] = 0.95, 95% confidence interval [CI]: 0.9182–0.9854, *p* < 0.01) and cereals (OR = 0.99, 95% CI: 0.9723–0.9982, *p* = 0.026) was associated with a reduced risk of depression, Whereas high tea consumption (OR = 1.01, 95% CI: 1.0009–1.0176, adjusted *p* = 0.029) was linked to an increased risk. Regarding anxiety disorders, non-oily fish intake (OR = 1.01, 95% CI: 1.0024–1.0121, *p* < 0.01) was positively associated with this risk, whereas unsalted peanuts (OR = 0.98, 95% CI: 0.9527–0.9986, *p* = 0.038) showed a protective effect.

**Conclusions:**

This MR study provides genetic evidence supporting the role of specific dietary exposures in influencing the risks of anxiety and depression. The findings highlight the potential of targeted dietary interventions in the prevention and management of mental health disorders.

## Introduction

Depression and anxiety are pervasive mental health disorders with major global public health implications. According to the World Health Organization, approximately 300 and 264 million people suffer from depression and anxiety disorders worldwide, respectively [1, 2, 3]. These conditions are linked to elevated risks of cardiovascular diseases, diabetes, immune dysfunction and suicide, collectively imposing severe emotional and societal burdens [4, 5, 6]. Hence, depression and anxiety impose a remarkably emotional burden and pose a direct threat to life.

Anxiety disorders, including generalised anxiety disorder, panic disorder, social anxiety disorder and specific phobias, are characterised by excessive fear and physiological hyperarousal. Major depressive disorder is marked by persistent low mood, anhedonia and cognitive and somatic symptoms [2]. Whilst the pathophysiology of these disorders involves complex neurochemical and neuroendocrine mechanisms, including monoamine neurotransmitter imbalances and hypothalamic–pituitary–adrenal (HPA) axis hyperactivation, emerging evidence highlights the important role of chronic inflammation and immune dysregulation [7, 8, 9].

Diet has been increasingly recognised as a modifiable factor influencing these biological pathways. Nutrients such as omega-3 fatty acids, vitamin D and B vitamins (e.g., B6, B12 and folate) are known to support neurotransmitter synthesis, reduce inflammation and maintain neuronal integrity, all of which are essential to mental well-being [10]. However, conventional observational studies exploring diet–mental health associations are often limited by residual confounding and reverse causation.

Mendelian randomisation (MR) has emerged as a robust method for assessing causal relationships using genetic variants as instrumental variables (IVs) to overcome the above limitations. MR minimises confounding and simulates the conditions of randomised trials [11, 12, 13]. This study applies a two-sample MR approach using large-scale European genome-wide association study (GWAS) data to evaluate the potential causal links between specific dietary exposures (e.g., vegetables, meat and fish) and the risks of anxiety and depression. The findings may inform future dietary interventions aimed at preventing and managing mental health disorders. Based on this rationale, it is hypothesized that a high intake of specific dietary components is causally associated with a reduced risk of depression and/or anxiety.

## Materials and Methods

### Study Design

**Fig. 1.**
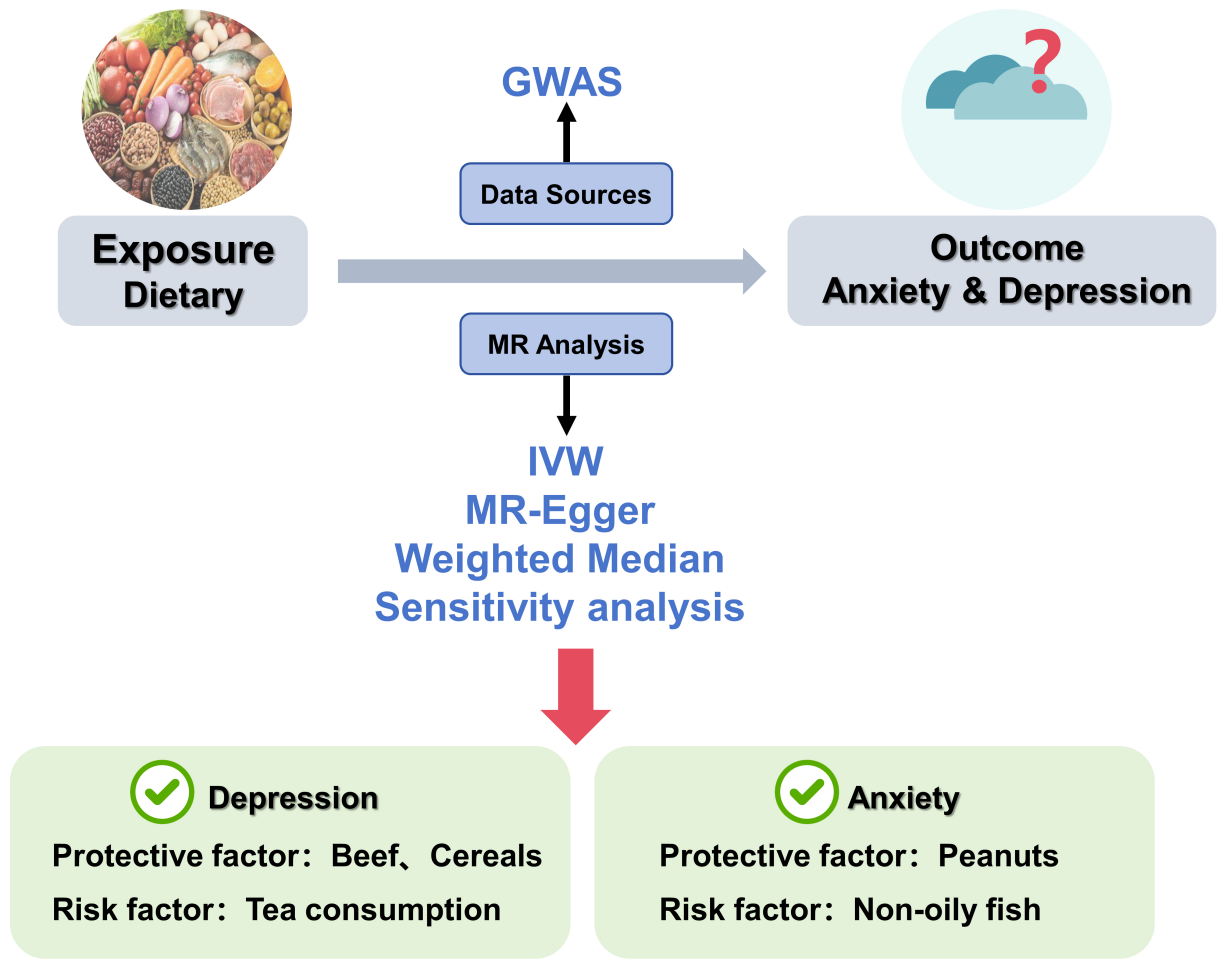
**Dietary impact on anxiety and depression: Mendelian randomisation (MR) analysis**. Genetic instruments from GWAS were used to evaluate dietary exposures. The inverse variance weighted (IVW) method was used as the primary MR analysis approach, together with MR–Egger and the weighted median method.

**Fig. 2.**
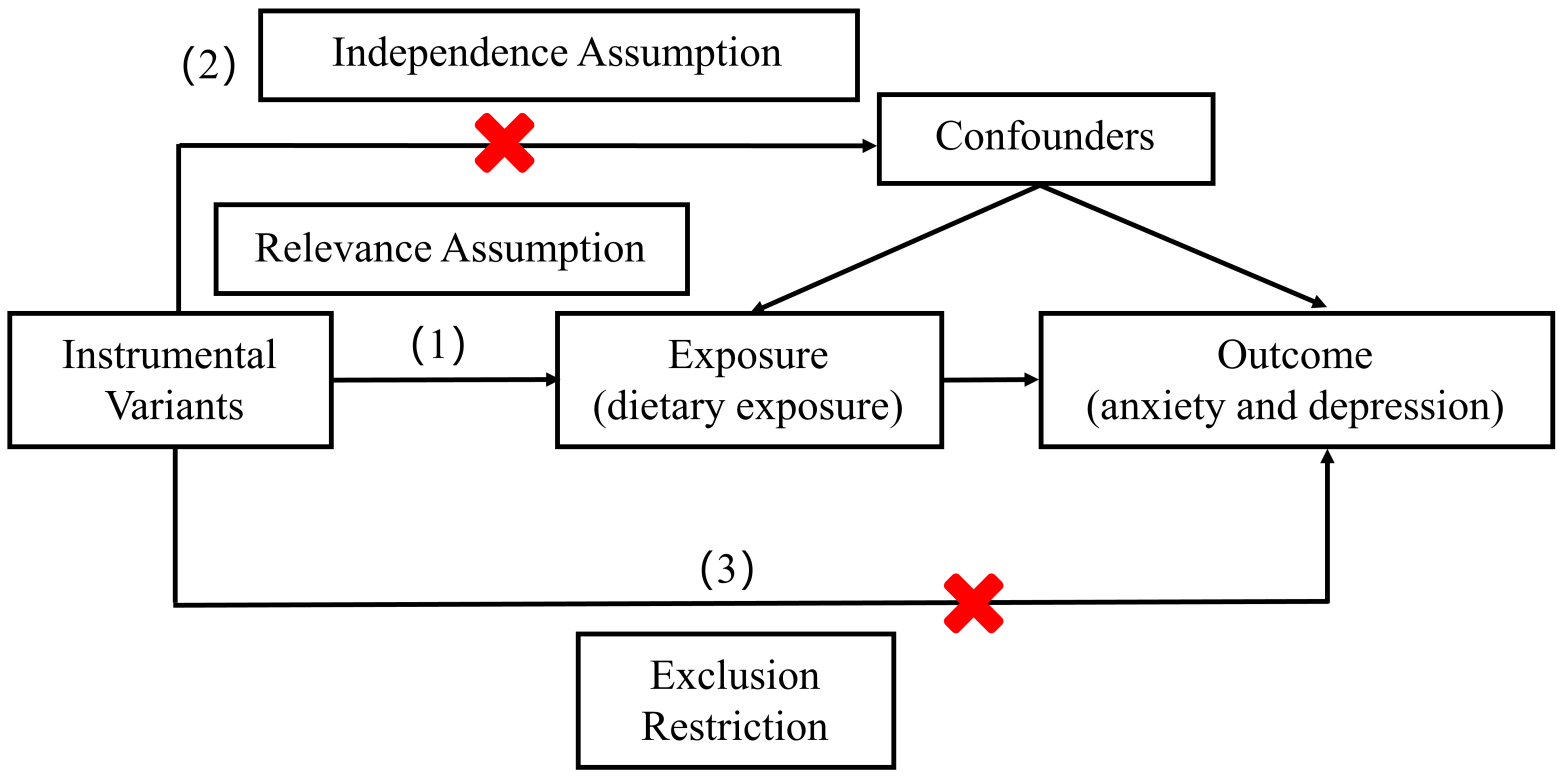
**Key assumptions of MR analysis**. This schematic illustrates the three core assumptions for valid MR inference: (1) the genetic instruments must be associated with the exposure (relevance assumption), (2) they must be independent of confounders (independence assumption) and (3) they must affect the outcome only through the exposure (exclusion restriction assumption).

This study employed a two-sample MR design to investigate the potential causal relationship between specific dietary exposures and the risks of anxiety and depression. MR is a method that uses genetic variants as IVs to infer causal relationships between an exposure (in this case, dietary exposures) and an outcome (anxiety and depression) without the need for experimental interventions (Fig. 1). To ensure the validity of the IVs, this study adhered to the following three core MR assumptions: (1) relevance assumption, which requires that the selected single nucleotide polymorphisms (SNPs) are strongly associated with the exposure (dietary exposures); (2) independence assumption, which stipulates that the selected SNPs are independent of confounders that could influence the outcome; and (3) exclusion restriction assumption, which ensures that the SNPs influence the outcome only through the exposure, not through other pathways (Fig. 2).

### Data Sources and Selection of Genetic Instruments

The exposure and outcome summary statistics used in this study were obtained from the Integrative Epidemiology Unit (IEU) GWAS database (https://gwas.mrcieu.ac.uk/), which provides genotype and phenotype data primarily from individuals of European ancestry. Depression data were derived from a GWAS [14] comprising 27,568 depression cases and 457,030 controls (GWAS ID: ebi-a-GCST90038650). Anxiety disorder data were obtained from the Neale Lab UK Biobank analysis (GWAS ID: ukb-d-KRA_PSY_ANXIETY) of 1092 cases and 360,102 controls, also predominantly of European descent. The dietary exposures included a range of food and beverage intake variables, such as vegetable intake (raw and cooked), meat intake (pork, beef, lamb and bacon), processed meat intake, fish intake (oily and non-oily fish), staple food intake (bread and cereals), dairy intake (milk, yogurt and cheese), beverage intake (coffee and tea), alcohol intake (red wine and beer), fruit intake (fresh and dried fruit), nut intake (salted and unsalted nuts), peanut intake (salted and unsalted peanuts) and fatty acid intake (saturated and polyunsaturated fatty acids). Data on anxiety and depression were also obtained from the GWAS database, encompassing 361,194 samples for anxiety and 484,598 samples for depression. These data were used in compliance with the ethical standards of relevant institutional review boards, and informed consent was obtained from all the participants. SNPs were selected as IVs based on their strong association with the dietary exposures. The primary selection criterion was set as a *p*-value of <5 × 10^-8^ to ensure that the SNPs have a strong statistical association with the exposure. If the number of SNPs meeting this criterion was insufficient, the threshold was relaxed to a *p*-value of <5 × 10^-6^. SNPs with a linkage disequilibrium (LD) coefficient (r^2^) >0.001 were excluded to avoid bias due to LD. Additionally, the F-statistic was calculated for each set of IVs, with SNPs having an F-value >10 considered strong IVs, to reduce the likelihood of weak instrument bias (Table 1).

**Table 1.** **Data related to depression and anxiety**.

GWAS ID	Exposure	n, case	n, control	Sample size	Number of SNPs	Source
ebi-a-GCST90038650	Depression	27,568	457,030	484,598	9,587,836	PUBMEDI: 33959723
ukb-d-KRA_PSY_ANXIETY	Anxiety disorders	1092	360,102	361,194	9,440,635	Neale lab

Note: This table summarises the genome-wide association study (GWAS) datasets used in the MR analysis of depression and anxiety outcomes. It includes the exposure type, number of cases and controls, total sample size, number of single nucleotide polymorphisms (SNPs) and source of each dataset.

### MR Analysis

MR analyses were conducted using the TwoSampleMR package (version 0.5.7; MRC Integrative Epidemiology Unit, University of Bristol, Bristol, UK) in R. The primary analytical methods included the inverse variance weighted (IVW) method, MR–Egger regression and weighted median method. IVW, which is the core method used in this study, estimates the overall causal effect of the dietary exposures on anxiety and depression by performing a weighted regression of the effect estimates from all the IVs. MR–Egger regression was employed to detect and adjust for pleiotropy, providing a corrected causal effect estimate in cases where the SNPs might influence the outcome through pathways other than the exposure. The weighted median method was used as a robust estimation technique that remains valid even if some of the IVs are invalid, as long as at least 50% of the weight in the analysis comes from the valid IVs.

### Sensitivity Analysis 

Several sensitivity analyses were performed to ensure the robustness of the MR results. Leave-one-out analysis was used to systematically exclude each IV one at a time, recalculating the meta-effect of the remaining SNPs to observe any significant changes in the results. If the exclusion of a specific SNP led to a substantial change in the results, that SNP was considered pleiotropic and excluded from the analysis. Additionally, the MR pleiotropy residual sum and outlier method was used to detect and correct for pleiotropy by identifying and removing outliers. The presence of heterogeneity among the IVs was assessed using Cochran’s Q statistic and the I^2^ statistic. If heterogeneity was detected (Q_*p*val >0.05), then the IVW random-effects model or the weighted median method was used; otherwise, the IVW fixed-effects model was applied.

### Statistical Analysis

All statistical analyses were performed using R software (version 4.2.1; R Foundation for Statistical Computing, Vienna, Austria). Causal relationships were considered robust if the results from the IVW, MR–Egger and weighted median methods were consistent, and the *p*-value from the IVW method was <0.05. Original *p*-values were adjusted using the Benjamini–Hochberg method to control the false discovery rate (FDR) and account for multiple testing across 19 dietary exposures and two mental health outcomes. These FDR-corrected values were reported throughout the manuscript and figures as adjusted *p*-values, with adjusted *p* < 0.05 considered statistically significant. The results of the sensitivity analyses, including heterogeneity and pleiotropy tests, were used to further validate the robustness of the causal inferences. This structured approach of combining multiple MR methods and sensitivity analyses ensures the reliability and validity of the causal estimates between the dietary exposures and mental health outcomes.

## Results

### Dietary Exposures and Their Potential Causal Relationship With Depression

**Fig. 3.**
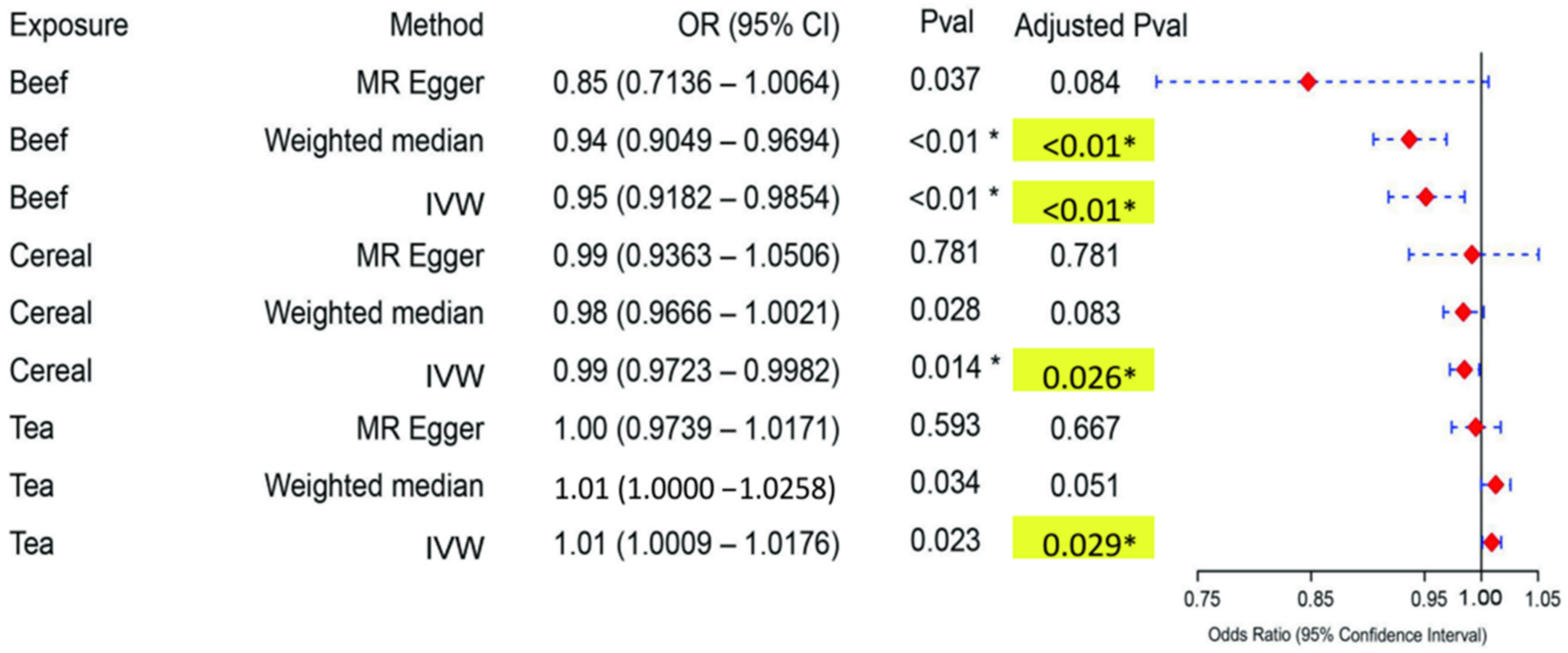
**Forest plot of the positive Mendelian randomisation results for the causal relationships between dietary exposures and depression risk**. This figure displays the causal effect estimates of various dietary exposures (beef, cereal and tea) on depression risk, derived from three Mendelian randomisation (MR) methods: MR–Egger, weighted median and inverse variance weighted (IVW). The odds ratios (ORs) and corresponding 95% confidence intervals (CIs) for each method are visualised, with red squares representing the ORs and horizontal lines indicating the 95% CI range. An OR <1 indicates a protective effect, whereas an OR >1 suggests an increased risk. Statistically significant results are highlighted in yellow, and *p*-values below 0.05 are marked with an asterisk (*). The reference line at OR = 1 indicates no effect, serving as a baseline for comparison. In this analysis, beef and cereal intake are associated with a reduced risk of depression (OR <1), suggesting their protective effects, whereas tea consumption is linked to an increased depression risk (OR >1). Statistical significance was determined using FDR-adjusted *p*-values (Benjamini–Hochberg correction), with adjusted *p* < 0.05 considered as significant.

**Fig. 4.**
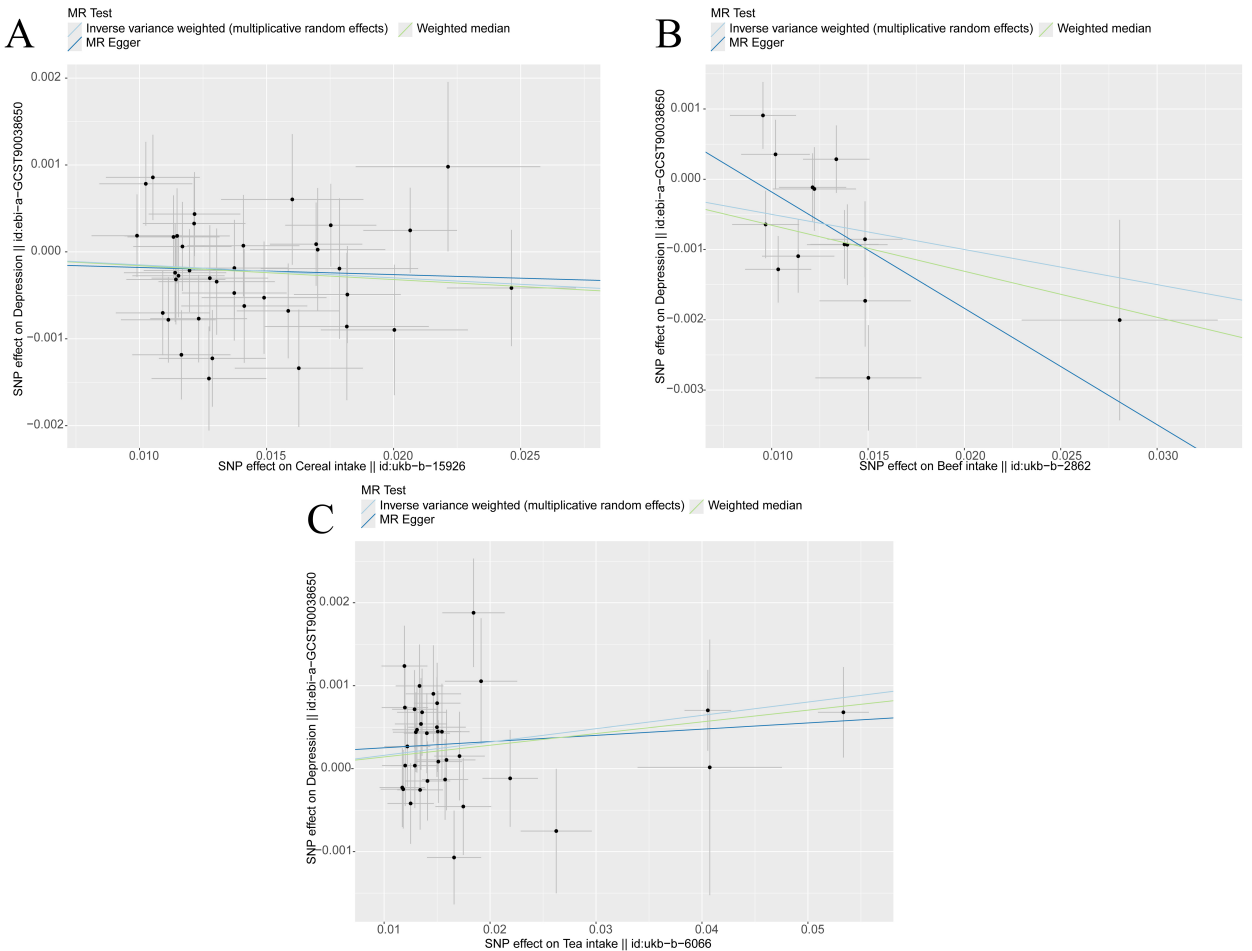
**Scatter plot of Mendelian randomisation analysis results, indicating the potential associations between various dietary exposures (cereal, beef and tea) and depression risk**. Each point represents a single nucleotide polymorphism (SNP). The different lines depict the regression slopes estimated by various MR methods (MR–Egger, weighted median and inverse variance weighted), illustrating the genetic associations between the exposures and outcomes. (A) Cereal and depression: Cereal intake and depression risk exhibit a negative correlation, that is, depression risk decreases with the increase in cereal consumption, suggesting a protective effect. (B) Beef and depression: A significant negative association is observed between beef consumption and depression risk, where the negative slope of the regression line indicates that a high beef intake corresponds to a reduced depression risk. (C) Tea and depression: Tea consumption shows a positive correlation with depression risk, with the upward slope suggesting that depression risk rises with tea intake. The error bars reflect the standard errors of the effect estimates, indicating the precision of the associations.

Depression is a prevalent mental health disorder with profound effects on global quality of life and social functioning. Identifying modifiable risk factors, such as dietary habits, may offer critical insights for developing prevention and treatment strategies. **Supplementary Fig. 1** shows the causal relationship between specific dietary exposures and depression risk. IVW analysis demonstrated a statistically significant inverse association between beef consumption and depression risk, with an OR of 0.95 (95% confidence interval [CI] 0.9182–0.9854, adjusted *p* < 0.01). The beta direction was consistent across IVW, weighted median and MR–Egger methods, indicating that increased beef consumption is associated with a reduced depression risk. For each standard deviation (SD) increase in beef consumption, the risk of depression decreases by approximately 4.9%, suggesting that beef may act as a protective factor against depression. Similarly, IVW analysis identified a significant negative association between cereal consumption and depression risk, with an OR of 0.99 (95% CI 0.9723–0.9982, adjusted *p* = 0.026). Although the beta direction was consistent across all three methods, the strength of the association appeared more prominent in the IVW analysis, whilst the other methods provided supporting trends without statistical significance. This pattern suggests the potential protective role of cereal, though further investigation is warranted. By contrast, IVW analysis revealed a statistically significant positive association between tea consumption and depression risk, with an OR of 1.01 (95% CI 1.0009–1.0176, adjusted *p* = 0.029). The consistent beta direction across IVW, weighted median and MR–Egger analyses suggests a potential association between increased tea consumption and high depression risk; however, only the IVW results reached statistical significance. For each SD increase in tea consumption, the risk of depression increases by approximately 0.9%, suggesting that tea may contribute to an elevated depression risk. These results suggest that beef and cereal consumption may offer protective effects against depression, whereas tea consumption may increase the risk. The consistency of these findings across multiple analytical methods underscores their robustness and provides a strong foundation for future research on the role of dietary exposures in depression prevention and treatment (Figs. 3,4). Whilst some observed ORs (e.g., OR = 0.95 for beef and OR = 0.99 for cereals) indicate modest reductions in depression risk, even small effect sizes may have meaningful implications at the population level, especially given the high prevalence of depression and the modifiable nature of dietary habits.

### Dietary Exposures and Their Potential Causal Relationship With Anxiety Disorders

**Fig. 5.**
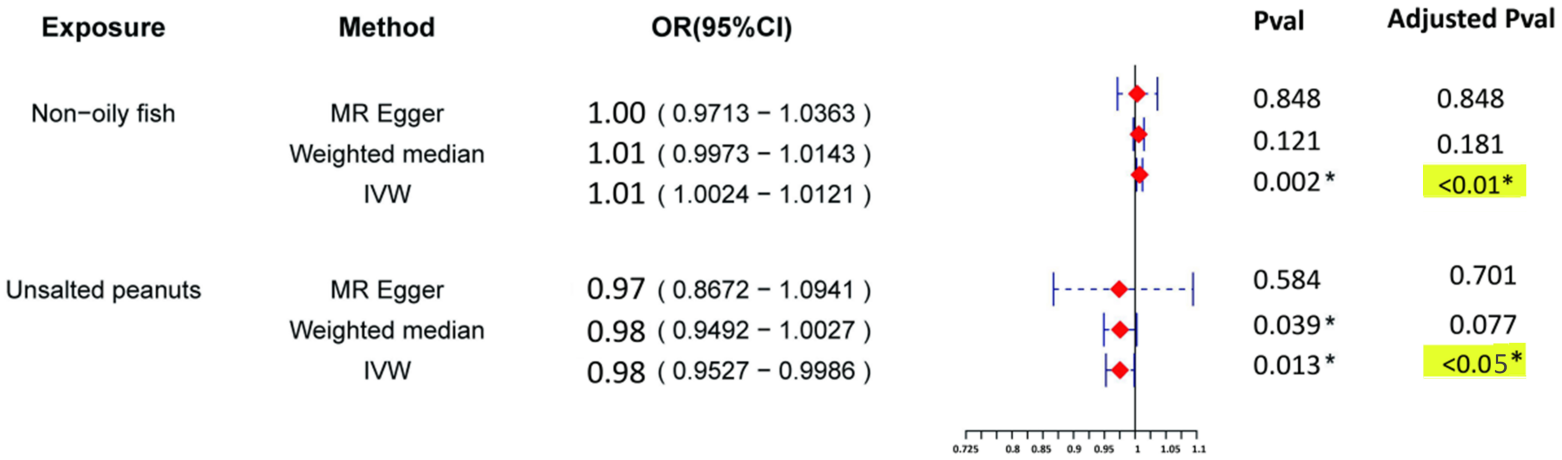
**Forest plot of MR results on anxiety disorders and dietary exposures**. The figure presents the odds ratios (ORs) and 95% confidence intervals (CIs) estimated using three Mendelian randomisation (MR) methods: MR–Egger, weighted median and inverse variance weighted (IVW). Red squares indicate the point estimates (ORs), and horizontal lines represent the corresponding 95% CIs. An OR >1 implies an increased risk of anxiety disorders, whereas an OR <1 indicates a potential protective effect. Statistical significance was determined using FDR-adjusted *p*-values (Benjamini–Hochberg correction), with adjusted *p* < 0.05 considered as significant. Statistically significant associations are marked with an asterisk (*) and highlighted in yellow for visual emphasis.

**Fig. 6.**
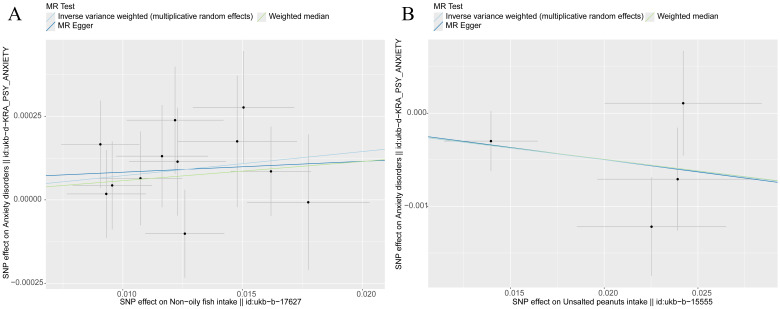
**Scatter plot of MR analysis results, indicating the potential associations between specific dietary exposures (non-oily fish and unsalted peanuts) and risk of anxiety disorders**. Each point represents a single nucleotide polymorphism (SNP). The different lines depict the regression slopes estimated by various Mendelian randomisation (MR) methods: MR–Egger, weighted median and inverse variance weighted (IVW), illustrating the genetic associations between the exposures and outcomes. (A) Non-oily fish and anxiety disorders: The upward trend in the regression lines indicates that non-oily fish intake and anxiety disorders show a slight positive correlation, suggesting that an increased non-oily fish consumption may be associated with a high risk of anxiety disorders. (B) Unsalted peanuts and anxiety disorders: A significant negative association is observed between unsalted peanut consumption and anxiety disorders, where the downward slope of the regression line indicates that a high unsalted peanut intake corresponds to a reduced risk of anxiety disorders. The error bars reflect the standard errors of the effect estimates, indicating the precision of the associations.

Anxiety disorders are prevalent mental health conditions that significantly impair daily functioning and psychological well-being. Identifying the potential causal influences of dietary exposures on anxiety disorders can provide crucial insights for developing effective prevention and management strategies. This section presents the results of MR analysis on the causal relationships between specific dietary exposures and risk of anxiety disorders. IVW analysis demonstrated a statistically significant positive association between non-oily fish consumption and risk of anxiety disorders, with an OR of 1.01 (95% CI 1.0024–1.0121, adjusted *p* < 0.01). Although the effect directions across MR–Egger and weighted median methods were consistent, their estimates were less pronounced. These results indicate a possible link between high non-oily fish consumption and increased anxiety risk, which may reflect specific nutrient composition or environmental factors associated with this food type. The beta direction was consistent across IVW, weighted median and MR–Egger analyses, indicating that a high non-oily fish consumption is associated with an increased risk of anxiety disorders. For each SD increase in non-oily fish consumption, the risk of anxiety disorders rises by approximately 0.73%, suggesting that non-oily fish may contribute to an elevated anxiety risk. Conversely, IVW analysis revealed a significant negative association between unsalted peanut consumption and anxiety risk, with an OR of 0.98 (95% CI 0.9527–0.9986, adjusted *p* = 0.038). The effect directions from other methods were aligned but less strong. This finding suggests the potential role of unsalted peanuts in reducing anxiety risk, possibly due to their micronutrient content and anti-inflammatory properties. Overall, these findings provide preliminary evidence that dietary exposures such as non-oily fish and unsalted peanuts may be involved in anxiety regulation. Whilst the strength of associations varied across the analytical approaches, the consistent effect directions support further exploration of these relationships in future studies (Figs. 5,6).

## Discussion

This study employed MR to systematically evaluate the potential causal relationships between various dietary exposures and the risks of anxiety and depression. The findings indicate that high consumption of beef and whole grains may be associated with a reduced depression risk, whereas increased tea intake could be linked to a high risk. Regarding anxiety disorders, non-oily fish consumption is positively correlated with depression risk, whereas unsalted peanuts might offer a protective effect. The inverse association between beef consumption and depression risk may be attributed to beef’s rich content of essential nutrients, notably tryptophan, vitamin B12, iron and zinc. Tryptophan serves as a precursor to serotonin, a key neurotransmitter in mood regulation [15, 16]. Vitamin B12 acts as a coenzyme in methylation reactions vital for the synthesis of monoamine neurotransmitters, such as dopamine, norepinephrine and serotonin [17]. Iron and zinc play crucial roles in the metabolism of dopamine and serotonin, maintenance of synaptic plasticity and regulation of neurotransmitter release and signal transduction [18]. Additionally, zinc modulates neuroinflammation and the expression of brain-derived neurotrophic factor, which is significant in the pathophysiology of depression [19]. Iron deficiency may lead to dopaminergic dysfunction, potentially triggering mood disorders [20]. These neuroregulatory nutrients in beef may synergistically enhance neurotransmitter balance and neural network function, thereby mitigating depression risk.

Whole grains, particularly in their unrefined form, are abundant in dietary fibre, B vitamins (such as folate and vitamin B6) and antioxidants, all of which may confer antidepressant effects through multiple mechanisms. Dietary fibre helps stabilise blood glucose levels, reduces mood fluctuations and influences the gut microbiota, thereby modulating the gut–brain axis and the HPA axis stress response and indirectly affecting mood regulation [21]. B vitamins are essential for neurotransmitter synthesis; for instance, folate deficiency can elevate homocysteine levels, thus impairing neuronal function and inducing depression [22]. Vitamin B6 is necessary for the synthesis of gamma-aminobutyric acid, a primary inhibitory neurotransmitter whose deficiency has been linked to depression [23]. The synergistic effects of these components in whole grains may support brain metabolism and provide neuroprotective benefits.

The positive association between tea consumption and depression risk may be related to the neuroactive properties of caffeine. Although caffeine can enhance alertness in the short term, excessive long-term intake may disrupt sleep patterns, increase anxiety and induce chronic stress [24]. Caffeine activates the HPA axis, elevates cortisol levels and may impair hippocampal function through disrupted negative feedback mechanisms, mirroring the HPA axis hyperactivity observed in depression [25]. Individual variations in caffeine metabolism could also lead to mood fluctuations and dependence symptoms in sensitive populations [26]. Although tea contains antioxidants such as polyphenols, their interactions with caffeine are not fully understood; high consumption warrants further investigation into its neuropsychiatric effects [27].

The observed association between non-oily fish consumption and increased anxiety risk is unexpected, given the general perception of fish as beneficial for mental health because of their omega-3 fatty acid content. Non-oily fish typically have low levels of omega-3, which may diminish their neuroprotective and anti-inflammatory effects. Additionally, certain non-oily fish species may accumulate environmental pollutants, such as mercury, which exert neurotoxic effects, disrupt neurotransmitter synthesis and release and potentially exacerbate anxiety symptoms [28, 29]. This finding underscores the need for further research into the specific types, preparation methods and contaminant exposure levels of fish for consumption to accurately assess their impact on mental health. Meanwhile, unsalted peanuts are rich in healthy fats, proteins and micronutrients beneficial for brain function, supporting their potential protective role against anxiety. The magnesium in peanuts can modulate the HPA axis, influencing the body’s stress response. Low magnesium levels are associated with heightened anxiety, and adequate magnesium intake from foods such as peanuts may help alleviate stress responses and reduce anxiety symptoms [30]. The niacin (vitamin B3) in peanuts also supports neural function and participates in neurotransmitter synthesis, potentially enhancing their anxiolytic effects [31]. These findings align with previous observational studies linking diets rich in whole grains, high-quality proteins and healthy fats to low incidences of depression and anxiety [32]. However, traditional observational studies are prone to confounding and reverse causality. By applying MR, the present work mitigates these issues and enables robust causal inference. Different from earlier MR studies that focused on single nutrients or food groups, the current research comprehensively evaluates multiple dietary exposures, advancing our understanding of the link between diet and mental health.

The following three MR methods were used to ensure result robustness: IVW, MR–Egger regression and weighted median. IVW provides the greatest statistical power but is sensitive to horizontal pleiotropy. MR–Egger detects directional bias but has low precision. The weighted median offers reliable estimates even when some instruments are invalid. The results are primarily based on IVW and supported by consistent findings across all methods and sensitivity analyses, reinforcing this study’s credibility.

Although the absolute effect sizes observed in this study are relatively small, the consistency across the methods and statistical significance supports their potential biological and public health relevance. In nutritional epidemiology, even modest shifts in risk can be impactful when translated into dietary recommendations for large populations.

Despite the strengths of this study, several limitations must be acknowledged. Firstly, the sample population predominantly comprises individuals of European descent, which may limit the generalisability of the findings to other ethnic groups with different genetic backgrounds, dietary habits and environmental exposures. Secondly, though MR methods effectively control for confounding and reverse causality, they cannot completely eliminate residual confounding, particularly from lifestyle factors such as physical activity, smoking and alcohol consumption. Variations in dietary practices and genetic structures across populations, such as high tea consumption in Asian countries with different tea types and preparation methods, may also influence metabolic pathways and the effectiveness of genetic instruments in MR analyses. Future research should aim to develop population-specific genetic instruments, conduct parallel analyses across diverse cohorts and employ stratified and multi-cohort MR approaches to enhance the applicability and interpretability of the results to the global population. Thirdly, this study focused on the independent effects of individual dietary exposures without exploring potential interactions between different foods. For instance, whether high tea consumption combined with low grain intake exerts additive or synergistic effects on mental health remains unclear. Future studies should investigate these complex dietary interactions to inform nuanced nutritional interventions. Additionally, shifting the research focus from single nutrients or foods to overall dietary patterns, such as the Mediterranean diet, may provide an accurate representation of real-world eating behaviours and their cumulative impact on mental health. Such holistic approaches could offer effective strategies for the prevention and management of mood disorders.

In summary, this study underscores the critical role of diet in mental health, revealing that specific dietary exposures have significant potential to influence the risks of anxiety and depression. The protective effects of beef and cereals against depression and the benefits of unsalted peanuts in reducing anxiety open promising avenues for dietary interventions. These findings provide a scientific foundation for developing targeted nutritional strategies to support mental health, emphasising the potential of dietary choices as a valuable tool in preventing and managing anxiety and depression.

## Conclusions

This study employed MR to explore potential causal links between dietary exposures and the risks of anxiety and depression, providing novel genetic evidence to support the role of diet in mental health. The findings suggest that dietary modification may represent a promising strategy for the prevention and management of mental disorders and warrant further investigation in broad populations.

## Consent of Publication

Not applicable.

## Availability of Data and Materials

The datasets used and/or analyzed during the current study are available from the corresponding author on reasonable request.

## Supplementary Material

Supplementary material associated with this article can be found, in the online version, at https://doi.org/10.62641/aep.v53i5.1969.
