# Supplementary material for: Mendelian Randomisation Analysis of Dietary Exposures and Potential Risks of Anxiety and Depression
Source: Actas Esp Psiquiatr. 2025 Oct 5;53(5):1093–103. doi: 10.62641/aep.v53i5.1969 (PMC12538602; doi:10.62641/aep.v53i5.1969)
Supplement: Supplementary file 1 [file ActEsp-53-5-1093-1103-s1.zip › Supplementary Fig. 1.docx]

**
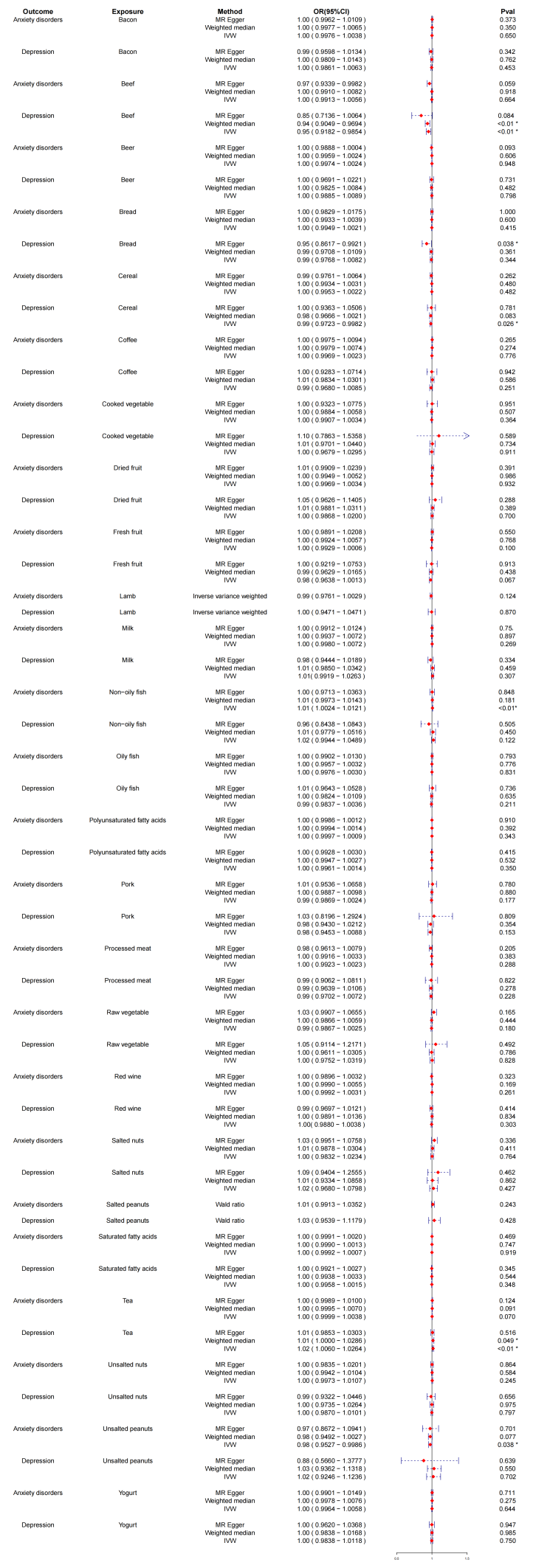
**

**Supplementary Fig. 1. The detailed results from the MR analysis for all identified markers and their associations with GI tumors.** GI, gastrointestinal; IVW, inverse variance weighted; MR Egger, mendelian randomization egger; CI, confidence interval; SNP, single nucleotide polymorphism. *p*-values below 0.05 are marked with an asterisk (*).
